# Aloe-Emodin Induces Breast Tumor Cell Apoptosis through Upregulation of miR-15a/miR-16-1 That Suppresses BCL2

**DOI:** 10.1155/2020/5108298

**Published:** 2020-03-03

**Authors:** Xuefeng Jiang, Yusheng Liu, Guijuan Zhang, Shujun Lin, Jieyan Wu, Xianxin Yan, Yi Ma, Min Ma

**Affiliations:** ^1^College of Traditional Chinese Medicine, Jinan University, Guangzhou, Guangdong 510632, China; ^2^The First Affiliated Hospital of Jinan University, Guangzhou, Guangdong 510630, China; ^3^Institute of Biomedicine and Department of Cellular Biology, Jinan University, Guangzhou, Guangdong 510632, China

## Abstract

**Purpose:**

Aloe-emodin (AE) is a natural compound derived from aloe vera and palmatum rhubarb and shows anticancer activities in various cancers. Bcl-2 family is the main regulator of cell death or cell survival. This study describes the effects of AE on proliferation of breast tumor (BT) cells.

**Methods:**

MCF-10A, MCF-10AT, MCF-7, and MDA-MB-231 cell lines were exposed to AE. Cell proliferation and apoptosis were assessed by CCK-8 and flow cytometry. Protein levels were measured by Western blotting. The levels of mRNA and miRNA were examined by RT-PCR. Bioinformatics was applied to screen miRNAs that bind to 3′-UTR of mRNA.

**Results:**

The results showed that AE selective activity inhibited the proliferation and induced apoptosis of MCF-10AT and MCF-7 cells but exhibited no significant inhibition in MCF10A and MDA-MB-231 cells. Mechanistically, AE dose-dependently decreased the protein expression of Bcl-2 and Bcl-xl, while it increased Bax protein expression in MCF-10AT and MCF-7 cells. The levels of Bcl-xl and Bax mRNA were altered by AE treatment, which was consistent with the protein expression results. However, Bcl-2 mRNA levels were not affected in either cell line, suggesting that AE may modulate the protein translation of Bcl-2 through miRNAs. In all candidate miRNAs that bind to 3′-UTR of Bcl-2, miR-15a and miR-16-1 were dose-dependently downregulated by AE. Moreover, inhibition of miR-15a/16-1 could eliminate the inhibition of MCF-10AT and MCF-7 cells growth by AE and could reverse the downregulation of AE-induced Bcl-2 protein level.

**Conclusion:**

Our research provides an important basis that AE induces BT cell apoptosis through upregulation of miR-15a/miR-16-1 that suppresses BCL2.

## 1. Introduction

Breast cancer (BC) is a type of molecular heterogeneous cancer, which is the most frequently diagnosed malignancy in women and the leading cause of cancer death in women worldwide. Global cancer statistics 2018 estimated that there were 2.08 million new cases and 626,679 deaths in BC [1]. Moreover, China accounts for 12.2 percent of all newly diagnosed BC cases and 9.6 percent of all BC deaths worldwide [2]. Despite recent advances in early diagnosis and treatment, BC continues to threaten the health of women worldwide [3]. Therefore, there is an urgent need to clarify the relevant molecular mechanisms and find new treatments to inhibit the development of BC.

*MicroRNAs* (miRNAs) is a kind of small noncoding RNA (19-25 nucleotides of short RNA) [4], usually with 3′ translation of complementary sequences to regulate 3′-UTR of messenger RNA (mRNA) translation and stability [5]. It has been reported that miRNA is involved in the regulation of almost all intracellular signaling pathways and a series of biological processes, such as inflammation, cell cycle regulation, apoptosis, stress response, differentiation, and migration [6]. Its abnormal regulation plays a crucial role in the occurrence and development of tumors [7]. Studies have proved that miRNAs in the miR-15/16 family have anticancer effect. In malignant pleural mesothelioma, the downregulation of miR-15/16 is due to transcriptional inhibition of c-myc, mainly through the control of mir-15b/16-2 site [8]. MiR-16 inhibits the proliferation of bladder cancer by negatively regulating the expression of cyclin D1 [9]. By directly binding CCND1 3′-UTR, miR-15a and miR-16-1 inhibited CCND1 transcription, inducing apoptosis and cell cycle arrest in osteosarcoma [10]. And miR-15/16 act as tumor suppressors by directly targeting BCL2 [11]. MiR-15/16 play a vital part in the coordination and regulation of early differentiation of tumor cell proliferation, survival, and memory.

Aloe-emodin (AE) is a natural compound derived from aloe vera or palmatum rhubarb [12]. These compounds have historically been used as natural dyes, but recent studies have demonstrated their medicinal value, such as antibacterial, anti-inflammatory, antiviral, anticancer, and antiaging properties [13, 14]. Studies have shown that AE has antiproliferation effects and induces apoptosis [15]; it can inhibit cell proliferation in SW620 and HT29 colorectal cancer cell lines [16, 17]. Studies have also shown that mTORC2 is a target of AE, which strongly inhibits AKT activation caused by PTEN deficiency [18]. It has been reported that AE inhibits HER-2 expression and cell proliferation in BC cells with HER-2 overexpression [19]. However, whether AE can inhibit the expression of Bcl-2 by upregulating the expression of miR-15/16 in BT cells and thus induce apoptosis is unclear.

We have studied BT for many years. Here, we investigate the inhibition of AE on BT cells and the effect of miR-15a/16-1 on the cell apoptosis of BT with AE treatment. In this study, AE has an obvious effect on inducing apoptosis of BT cells. In addition, the downregulation of Bcl-2 protein and upregulation of miR-15a/16-1 may be related to AE-induced apoptosis.

## 2. Materials and Methods

### 2.1. Reagents and Chemicals

The AE was provided from National Institutes for Food and Drug Control (Guangzhou, China). Dulbecco's Modified Eagle Medium (DMEM), medium-nutrient mixture F-12 (DMEM/F12), horse serum (HS), fetal bovine serum (FBS), and epidermal growth factor (EGF) were purchased from Gibco BRL (Grand Island, NY, USA). Lipofectamine 2000 and Leibovitz's L-15 medium were purchased from Invitrogen Life Technologies (Carlsbad, CA, USA). Cell Counting Kit-8 (CCK-8) was purchased from Dojindo Molecular Technologies (Tokyo, Japan). Annexin V-FITC/Propidium Iodide (PI) Apoptosis Detection Kit was obtained from the BD Biosciences (San Jose, CA, USA). RNAiso Plus and PrimeScript RT reagent Kit with gDNA Eraser were obtained from Takara (Tokyo, Japan). All Bcl-2, Bcl-xl, Bax, and *β*-actin are derived from cell signaling techniques (Beverly, MA, USA).

### 2.2. Cell Culture and Transfection

MCF10A, MCF-7, and MDA-MB-231 cell lines were purchased from the American Type Culture Collection (ATCC, Manassas, VA, USA). MCF-10AT cell line was obtained from the Karmanos Cancer Institute (KCI). MCF-10A and MCF-10AT cells were cultured in DMEM/F12 containing 5% HS, 0.5 *μ*g/ml hydrocortisone, 100 ng/ml cholera toxin, 20 ng/ml recombinant human EGF, 10 *μ*g/ml insulin, and 1% penicillin/streptomycin. MCF-7 cells were maintained in DMEM supplemented with 10% FBS and 1% penicillin/streptomycin. MDA-MB-231 cells were cultured in L-15 medium supplemented with 10% FBS and 1% penicillin/streptomycin. All cells were grown in a humidified atmosphere containing 5% CO_2_ at 37°C.

The AE was dissolved in dimethyl sulfoxide (DMSO, Sigma) and diluted with culture medium to final concentrations before treatment. In all experiments, the final concentration of DMSO in AE medium solution was less than 0.1%, which had no effect on the cells.

miR-15a and miR-16-1 mimics and inhibitors were purchased from RiboBio (Guangzhou, China). According to the manufacturer's instructions, the cell lines were transfected with 100 nM of miR-15a, miR-16-1 or negative control using Lipofectamine 2000 (Invitrogen) in 6-well plates. Transfected cells were collected 24 h after transfection. Besides, NC-treated cells or untreated cells were used as a control group of the study.

### 2.3. Cell Proliferation Assay

The CCK-8 assay was performed to test cell proliferation. In short, cells were treated with various concentrations of AE in 96-well plates for 24, 48, and 72 h. 10 *μ*l of 5 mg/ml CCK-8 solution was added to each well for a further 2 h incubation. The optical density (OD) values were quantified by a microplate reader at 450 nm.

### 2.4. Apoptosis Analysis

To verify the effects of AE on the percentage of apoptosis, the cells were examined by Annexin V/PI apoptotic assay kit. The cells were harvested, washed, and resuspended in binding buffer. Then, the cells were stained with 2.5 *μ*l FITC and 2.5 *μ*l PI in the dark for 15 min. The apoptosis rate was determined using the flow cytometry.

### 2.5. Quantitative Real-Time PCR

After treatment of cells with AE, total RNA was extracted from cells using the RNAiso Plus. The total RNA concentration and purity were measured using micronucleic acid spectrophotometer. The cDNA was synthesized from total RNA according to the instructions of the PrimeScript RT reagent kit. Subsequently, according to the operation procedure, qRT-PCR was performed on Applied Biosystems 7900 real-time PCR system using SYBR Premix Ex Taq II with primers as shown in Table 1. The relative quantification results were calculated using the formula 2^–ΔΔCt^.

### 2.6. Western Blot Analysis

Total protein was extracted using the RIPA lysis buffer (Bio-Rad, Shanghai, China). Protein concentrations were quantified by BCA assay (Beyotime, Nanjing, China). The equal amounts of protein were separated by gel electrophoresis using 12% SDS-PAGE and transferred to PVDF membranes (Millipore, Darmstadt, Germany) for immunoblotting. After soaking in blocking buffer for 2 h, the primary antibody was incubated overnight at 4°C and then incubated with secondary antibody coupled with horseradish peroxidase. The labeled protein spots were detected by the gel automatic imaging system.

### 2.7. Statistical Analysis

The experimental data were analyzed using GraphPad Prism 6.0 software (GraphPad Software, Inc., La Jolla, CA, USA). All data were expressed as mean ± SD of at least three experiments. Student's *t*-test and one-way ANOVA with a Bonferroni correction were used to evaluate the quantitative data for statistical significance. *P* < 0.05 was considered statistically significant.

## 3. Results

### 3.1. AE Dose-Dependently Suppresses BT Cell Proliferation

**Figure 1:**
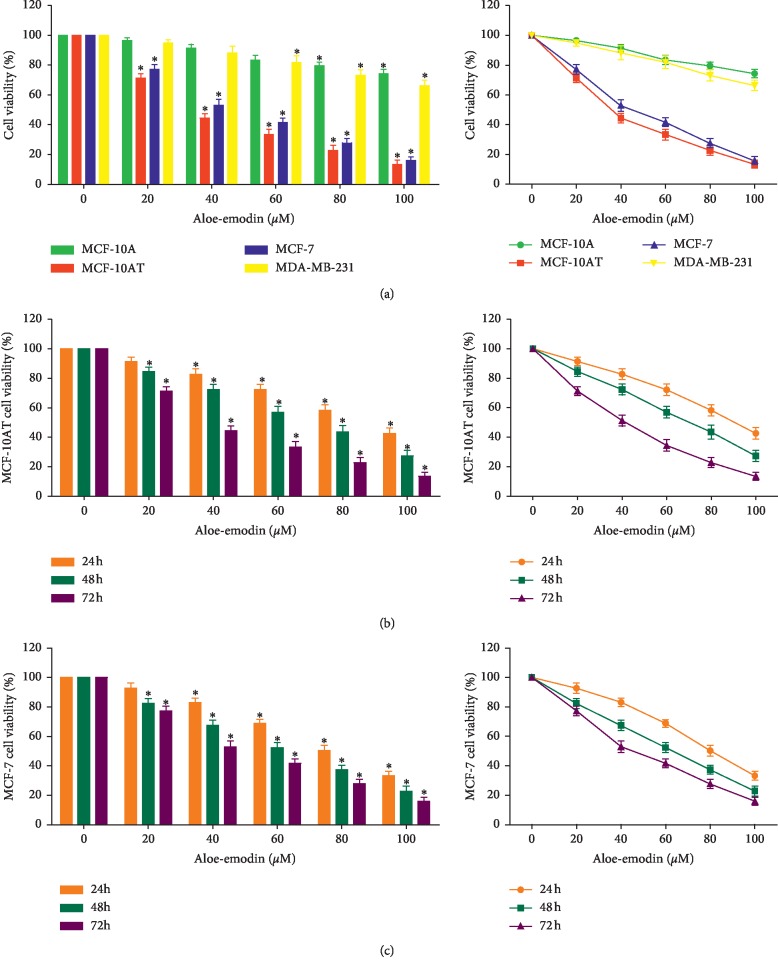
Effects of AE on cells proliferation. (a) MCF-10A, MCF-10AT, MCF-7, and MDA-MB-231 cells were treated with increasing doses of AE (0–100 *μ*M) for 72 h incubation. MCF-10AT (b) and MCF-7 (c) cells were treated with increasing doses of AE (0–100 *μ*M) for 24 h, 48 h, and 72 h incubation. The data of cells viability were shown as mean ± SD of three independent experiments, ^*∗*^*P* < 0.05.

The effects of AE (20, 40, 60, 80, and 100 *μ*M) on cell proliferation were examined in MCF-10A, MCF-10AT, MCF-7, and MDA-MB-231 cell lines using the CCK-8 assay. As shown in Figure 1(a), AE inhibited these cell lines' growth in a dose-dependent manner, and AE distinctly decreased the viability of MCF-10AT and MCF-7 cells in a time- and dose-dependent manner (Figures 1(b) and 1(c)). The 50% inhibitory concentrations (IC50) of AE on MCF-10AT and MCF-7 cells were about 35.75 *μ*M (72 h) and 43.49 *μ*M (72 h), respectively. While the effect of AE on MCF-10A and MDA-MB-231 cells were also evaluated, the IC50 of AE exceeded 100 *μ*M. Therefore, AE had a stronger inhibition on MCF-10AT and MCF-7 cells than on MCF-10A and MDA-MB-231 cells.

### 3.2. AE Induces Apoptosis in MCF-10AT and MCF-7 Cells

**Figure 2:**
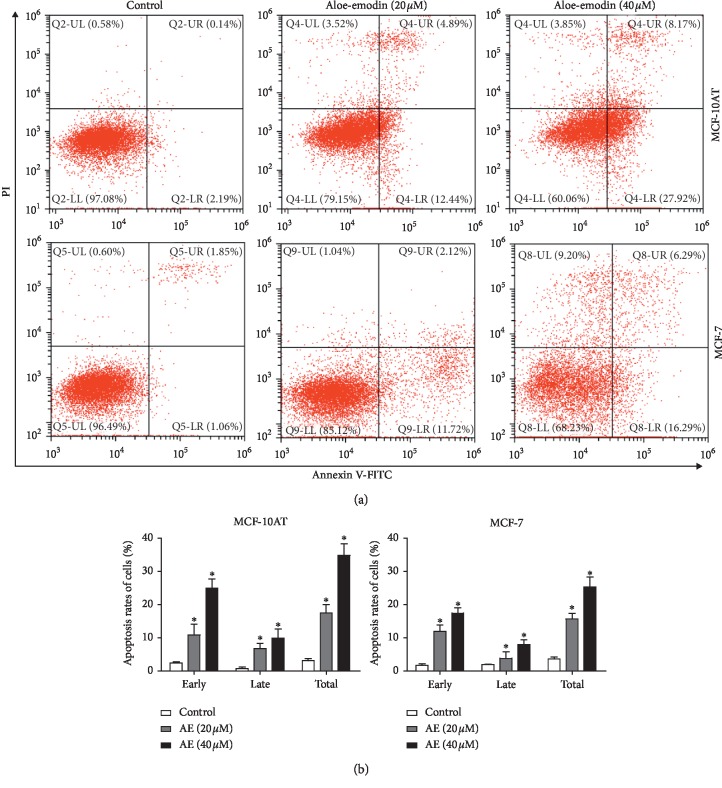
Effect of AE on apoptosis of MCF-10AT and MCF-7. (a) The cell apoptosis rates for AE-treated MCF-10AT and MCF-7 cells were assessed by FITC Annexin V/PI staining. (b) The MCF-10AT and MCF-7 cells in (a) apoptosis rates were quantified. The cell apoptosis rates were shown as the mean ± SD of three independent experiments, ^*∗*^*P* < 0.05.

MCF-10AT and MCF-7 cells were treated with PBS and AE (20 or 40 *μ*M) for 72 h and then the percentage of apoptosis cells was measured by Annexin V/PI staining assay. As shown in Figure 2, MCF-10AT cell apoptosis rates were 2.33%, 17.33%, and 36.09% in control, AE (20 *μ*M), and AE (40 *μ*M) groups. MCF-7 cell apoptosis rates were 2.91%, 13.84%, and 22.58% in control, AE (20 *μ*M), and AE (40 *μ*M) groups. The flow cytometry analysis indicated that the number of early and late apoptotic cells increased significantly after AE treatment.

### 3.3. AE Regulates Bcl-2, Bcl-xl, and Bax Expression in MCF-10AT and MCF-7 Cells

**Figure 3:**
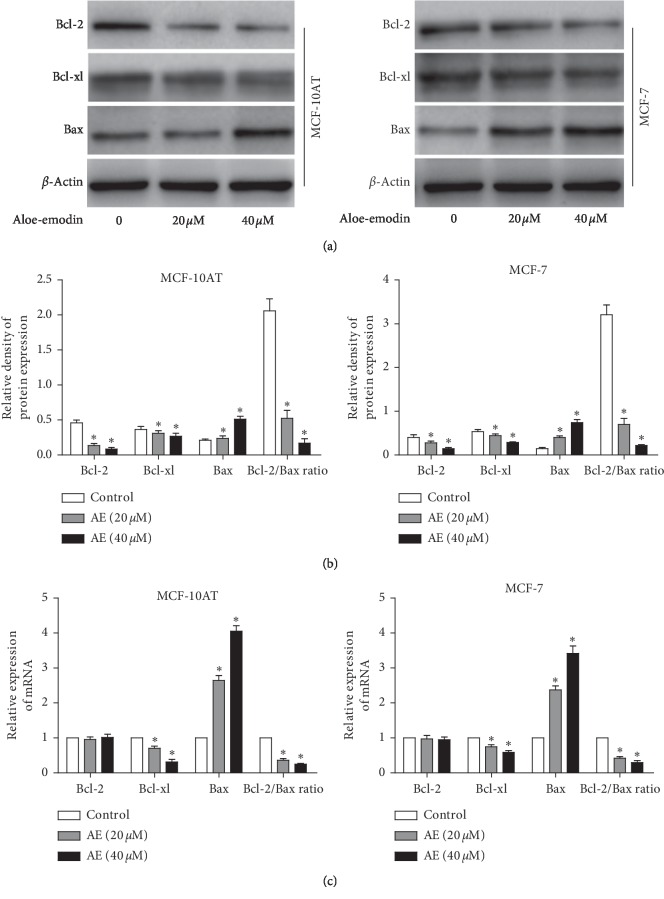
Effect of AE on the expression of Bcl-2, Bcl-xl, and Bax in MCF-10AT and MCF-7 cells. (a) Western blotting for Bcl-2, Bcl-xl, and Bax protein in MCF-10AT and MCF-7 cells treated with AE. (b) Relative proteins expression of Bcl-2, Bcl-xl, and Bax and the ratio of Bcl-2/Bax in (a). (c) The relative mRNAs expression of Bcl-2, Bcl-xl, and Bax and the ratio of Bcl-2/Bax after AE treatment in MCF-10AT and MCF-7 cells. The data were shown as mean ± SD of three independent experiments, ^*∗*^*P* < 0.05.

Bcl-2 family members play vital roles in the regulation of cell apoptosis progression by regulating antiapoptotic proteins, proapoptotic effectors, and proapoptotic activators [20]. In order to understand the underlying mechanisms, we examined protein levels of cell apoptosis regulators and found that the expression of Bcl-2 and Bcl-xl was dose-dependently decreased, while Bax expression was dose-dependently increased by AE in MCF-10AT and MCF-7 cells (Figures 3(a) and 3(b)). The RT-PCR results showed that AE treatment resulted in altered expression of Bcl-xl and Bax mRNA (Figure 3(c)). The mRNA and protein ratio of Bcl-2/Bax went down significantly (*P* < 0.05), indicating that AE induced apoptosis by reducing the ratio of Bcl-2/Bax in MCF-10AT and MCF-7 cells. However, the Bcl-2 mRNA levels were not affected in either line, suggesting that AE may modulate the protein translation of Bcl-2 through miRNAs.

### 3.4. miR-15a and miR-16-1 Target 3′-UTR of Bcl-2 mRNA to Suppress Its Protein Translation

**Figure 4:**
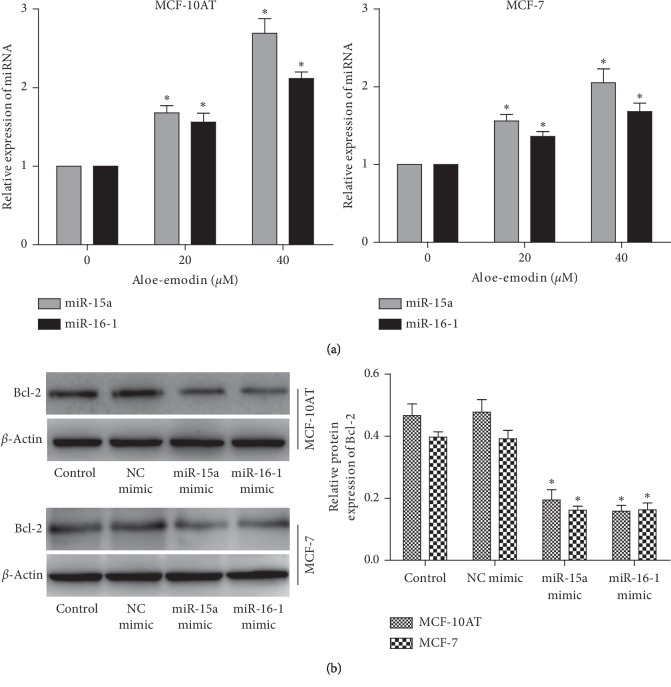
miR-15a and miR-16-1 bind to 3′-UTR of Bcl-2 mRNA to suppress its protein translation. (a) RT-qPCR for miR-15a and miR-16-1 levels in MCF-10AT and MCF-7 cells treated with AE. (b) The expression of Bcl-2 protein in MCF-10AT and MCF-7 cells transfected with miR-15a or miR-16-1 mimics. The data were shown as mean ± SD of three independent experiments, ^*∗*^*P* < 0.05.

We used bioinformatics tools to screen miRNAs that bind to 3′-UTR of Bcl-2 and found miR-15a and miR-16-1 as the candidates that were specifically and dose-dependently upregulated by AE (Figure 4(a)). Cimmino et al. demonstrated that Bcl-2 is the target of miR-15a and miR-16-1 [21]. Taking this into consideration, MCF-10AT and MCF-7 cells were transfected with plasmids carrying miR-15a or miR-16-1 mimics and the expression of Bcl-2 protein was measured by Western blot. The results showed that, compared with control group, Bcl-2 protein expression was decreased in the transfected miR-15a or miR-16-1 mimics group (Figure 4(b)).

### 3.5. Inhibition of miR-15a and miR-16-1 Eliminates the Inhibition of AE on MCF-10AT and MCF-7 Cells Growth

**Figure 5:**
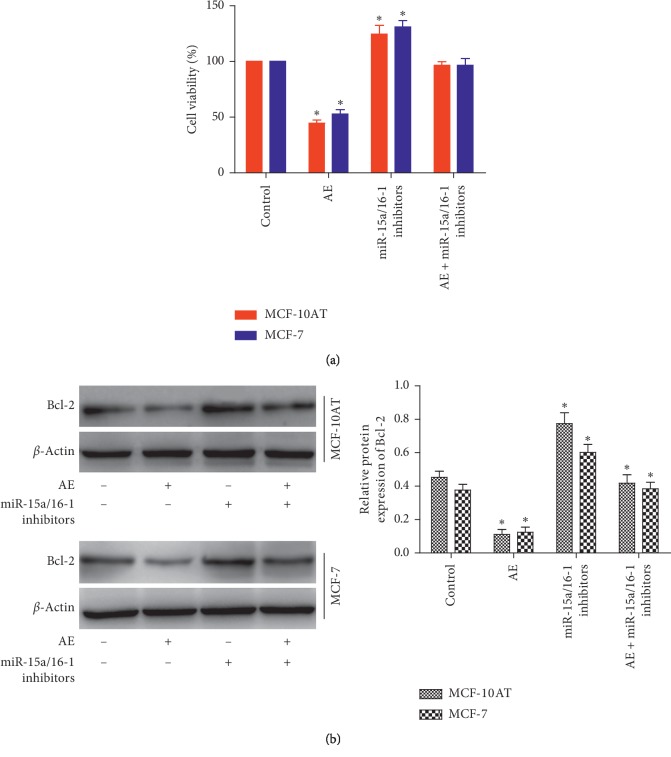
AE inhibits MCF-10AT and MCF-7 cells growth through upregulation of miR-15a/16-1 that suppresses Bcl-2. (a) Inhibition of miR-15a/16-1 eliminates the inhibition of AE on MCF-10AT and MCF-7 cells growth. (b) The expression of Bcl-2 protein in MCF-10AT and MCF-7 cells with different treatments were measured by Western blot. The data were shown as mean ± SD of three independent experiments, ^*∗*^*P* < 0.05.

To investigate whether the inhibition of AE on MCF-10AT and MCF-7 cells growth was mediated by alteration in miR-15a/16-1 suppressed Bcl-2 expression, we exposed MCF-10AT and MCF-7 cells that downregulated miR-15a/16-1 with AE. First, cell viability was detected in MCF-10AT and MCF-7 cells transfected with miR-15a/16-1 inhibitors. We found that inhibition of miR-15a/16-1 eliminates the inhibition of AE on MCF-10AT and MCF-7 cells growth (Figure 5(a)). Next, we measured Bcl-2 protein levels in each group. Compared with the control group, the proteins expression of Bcl-2 was decreased in AE-treated group (*P* < 0.05). The miR-15a/16-1 were downregulated, which could reverse the downregulated proteins levels of Bcl-2 induced by AE (Figure 5(b)).

## 4. Discussion

AE (1,8-dihydroxy-3-(hydroxymethyl)anthracene-9,10-dione), as an anthraquinone derivative of phytoestrogens, can be obtained from the root and rhizome of rhubarb and the leaves of aloe vera, and its antitumor activity has been reported in many previous studies. Mildred et al. showed that AE can regulate PKC isoenzyme, inhibit proliferation, and induce U-373MG glioma cell apoptosis [15]. It has also been shown that AE can induce apoptosis in nasopharyngeal carcinoma cell lines (NPC-TW 039 and NPC-TW 076 cells) by regulating caspase activities, including caspase-3, caspase-8, and caspase-9 [22]. Chang et al. found that AE inhibits the ERK- and AKT-related signaling pathways activated to suppress the proliferation of esophageal cancer cell TE1 [23]. Huang et al. indicated that AE is capable of inhibiting BC cell proliferation by suppressing ERα transcriptional activation [24].

Here, we used MCF10A, MCF-10AT, MCF-7, and MDA-MB-231 cell lines to assess the role of AE in regulating BT cell proliferation. The human cell line MCF-10A originated from spontaneous immortalization of breast epithelial cells from a patient with fibrocystic disease. The cell has the characteristics of nontumorigenic “normal” breast epithelium. MCF-10A cells did not survive in vivo in immunodeficient mice. However, the MCF-10A cells transfected with T24 c-Ha-ras oncogene (MCF-10AT cells) formed small nodules in nude mice and occasionally developed into carcinomas. A significant number progressed to lesions resembling atypical hyperplasia and carcinoma in situ in women, and approximately 25% progressed to invasive carcinomas, representing the transformation of normal epithelial cells to malignant tumors [25, 26]. MCF-7 cell line is luminal estrogen receptor-positive BC cell line, and the triple negative MDA-MB-231 cell line is also selected for this study. The different characteristics of the four cell lines make our results more useful and applicable to general BT study and treatment. We found that AE can inhibit breast tumor cells proliferation markedly in a dose- and time-dependent manner. At 72 hours, the IC50 of AE on MCF-10AT and MCF-7 cells was about 35.75 *μ*M and 43.49 *μ*M, respectively. However, the same concentration of AE did not have significant effect on the MCF-10A and MDA-MB-231 cells. Huang's research indicated that the inhibitory effects of AE on the growth of ER-negative MDA-MB-453 cell were moderate compared to the effects on ER-positive MCF-7 cells, and ER*α* protein played an important role in the AE-induced suppression of breast cancer cell proliferation [24]. Meanwhile, Cittelly's study found that miR-15a/16 is less expressed and cannot regulate the expression of bcl-2 protein and affect cell apoptosis in MDA-MB-231 cells [27]. Our study is consistent with previous studies. Hence, we decided to explore the effect and mechanism of AE on MCF-10AT and MCF-7cells.

The regulation of cell proliferation depends on the balance between cell division and cell death. Apoptosis is a kind of independent-like programmed cell death [28]. AE can induce apoptosis in various cancer cells [29, 30]. In this study, the flow cytometry analysis showed that the number of early and late apoptotic BT cells increased significantly after AE intervention. Bcl-2 family members are main regulators of cell death or cell survival. Tumor cells routinely violate cellular checkpoints in normal cells that initiate cell death by triggering proapoptotic members of the BCL-2 family of proteins. In order to avoid this death-inducing signals, tumor cells are often selected for upregulation of antiapoptotic BCL-2 family members including BCL-2, BCL-xl, BCL-W, BFL-1, and MCL-1 [31]. In contrast, proapoptotic BCL-2 family members are actively involved in inducing cell death, such as Bax and Bak [32]. Abnormal high expression of Bcl-2 protein promotes the growth of BT; Bcl-2 and Bax constitute the apoptotic switch in tumorigenesis and treatment [33]. Bcl-xl is a strong antiapoptotic member that heterodimerizes with Bax and neutralizes the effects of the latter [34]. Thus, Bcl-2 family members are potential targets for tumor therapy. In this study, we confirmed that AE dose-dependently decreased the protein expression of Bcl-2 and Bcl-xl, while it increased the Bax protein expression in MCF-10AT and MCF-7 cells. After AE treatment, the expression of Bcl-xl and Bax mRNA in MCF-10AT and MCF-7 cells was altered, which was consistent with the protein expression results. The mRNA and protein ratio of Bcl-2/Bax went down significantly, indicating that AE induced apoptosis by reducing the ratio of Bcl-2/Bax. However, Bcl-2 mRNA level was not affected in either line, which suggested that AE may regulate the levels of Bcl-2 at the posttranscriptional level through miRNAs.

**Figure 6:**
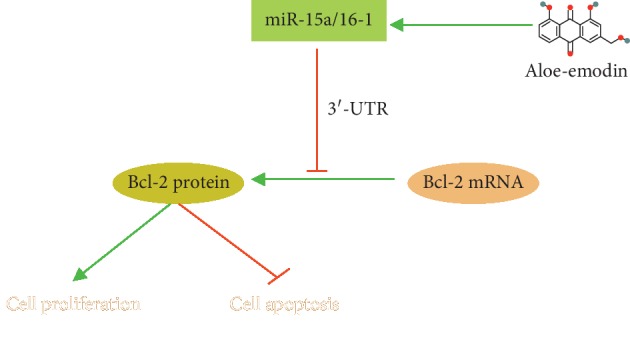
Schematic diagram of the mechanism underlying this study. AE induces BT cell apoptosis through regulation of miR-15a/16-1/Bcl-2 signaling.

To investigate the mechanism of this regulation, we screened some miRNAs that bind to 3′-UTR of Bcl-2 by bioinformatics analysis and found miR-15a and miR-16-1 as the candidates that were specifically and dose-dependently downregulated by AE. Compared with the control group, the expression of Bcl-2 protein in the transfected miR-15a and miR-16-1 mimic group was decreased; the inhibition of miR-15a/16-1 could eliminate the inhibition of MCF-10AT and MCF-7 cells growth by AE and reverse the downregulation of AE-induced Bcl-2 protein level. All these indicated that AE decreased Bcl-2 to promote apoptosis of BT cells by regulating miR-15a/16-1 (Figure 6). However, the exact molecular regulation of AE on miR-15a/16-1 expression is unclear. AE may alter the expression of miR-15a/16-1 at transcriptional level, or indirectly alter them through hormones or cytokines. This interesting question may be solved in future studies.

## 5. Conclusion

In brief, this study shows that AE can effectively reduce or inhibit the development of BT by regulating miR-15a/16-1-induced apoptosis. Our research provides an important basis for further exploration of the possible application of AE in the prevention and treatment of BT.

## Figures and Tables

**Table 1:** Primers for quantitative real-time PCR.

Genes	Forward (5′∼3′)	Reverse (5′∼3′)
Bcl-2	GGTGGGGTCATGTGTGTGG	CGGTTCAGGTACTCAGTCATCC
Bcl-xl	GAGCTGGTGGTTGACTTTCTC	TCCATCTCCGATTCAGTCCCT
Bax	CCCGAGAGGTCTTTTTCCGAG	CCAGCCCATGATGGTTCTGAT
*β*-actin	CTACCTCATGAAGATCCTCACCGA	TTCTCCTTAATGTCACGCACGATT
miR-15a	CGGGCTAGCAGCACATAATG	CAGCCACAAAAGAGCACAAT
miR-16-1	CGGGCTAGCAGCACGTAAAT	CAGCCACAAAAGAGCACAAT
U6	CTCGCTTCGGCAGCACA	AACGCTTCACGAATTTGCGT

## Data Availability

Most of the data (Figures 1–6 and Table 1) used to support the findings of this study are included within the article. Other data that support the findings of this study are available from the corresponding author upon reasonable request.
